# Mast Cells Granular Contents Are Crucial for Deep Vein Thrombosis in Mice

**DOI:** 10.1161/CIRCRESAHA.117.311185

**Published:** 2017-07-24

**Authors:** Tatyana Ponomaryov, Holly Payne, Larissa Fabritz, Denisa D. Wagner, Alexander Brill

**Affiliations:** From the Institute of Cardiovascular Sciences, College of Medical and Dental Sciences, University of Birmingham, United Kingdom (T.P., H.P., L.F., A.B.); Department of Cardiology, University Hospital Birmingham, United Kingdom (L.F.); Program in Cellular and Molecular Medicine (D.D.W., A.B.) and Division of Hematology/Oncology (D.D.W., A.B.), Boston Children’s Hospital, MA; and Department of Pediatrics, Harvard Medical School, Boston, MA (D.D.W., A.B.).

**Keywords:** endothelium, histamine, mast cells, venous thrombosis

## Abstract

**Rationale:**

Deep vein thrombosis (DVT) and its complication pulmonary embolism have high morbidity reducing quality of life and leading to death. Cellular mechanisms of DVT initiation remain poorly understood.

**Objective:**

We sought to determine the role of mast cells (MCs) in DVT initiation and validate MCs as a potential target for DVT prevention.

**Methods and Results:**

In a mouse model, DVT was induced by partial ligation (stenosis) of the inferior vena cava. We demonstrated that 2 strains of mice deficient for MCs were completely protected from DVT. Adoptive transfer of in vitro differentiated MCs restored thrombosis. MCs were present in the venous wall, and the number of granule-containing MCs decreased with thrombosis. Pharmacological depletion of MCs granules or prevention of MC degranulation also reduced DVT. Basal plasma levels of von Willebrand factor and recruitment of platelets to the inferior vena cava wall after DVT induction were reduced in MC-deficient mice. Stenosis application increased plasma levels of soluble P-selectin in wild-type but not in MC-deficient mice. MC releasate elevated ICAM-1 (intercellular adhesion molecule-1) expression on HUVEC (human umbilical vein endothelial cells) in vitro. Topical application of compound 48/80, an MC secretagogue, or histamine, a Weibel–Palade body secretagogue from MCs, potentiated DVT in wild-type mice, and histamine restored thrombosis in MC-deficient animals.

**Conclusions:**

MCs exacerbate DVT likely through endothelial activation and Weibel–Palade body release, which is, at least in part, mediated by histamine. Because MCs do not directly contribute to normal hemostasis, they can be considered potential targets for prevention of DVT in humans.

## Abstract 2

Supplemental Digital Content is available in the text.

Venous thromboembolism, designating deep vein thrombosis (DVT) together with pulmonary embolism (PE), is a severe disease frequently diagnosed in the Western world. This pathological condition develops in ≈100 to 200 per 100 000 individuals annually.^1^ In a part of patients (≈69 per 100 000),^2^ DVT is complicated by PE, in which blood clot detaches and reaches lungs, leading to death in ≈30% of cases.^3^ As per estimation of the American Surgeon General, these diseases are responsible for >100 000 deaths a year in the United States only,^4^ exceeding mortality after myocardium infarction or stroke.^5^ Even after successful treatment, DVT and PE may result in post-thrombotic syndrome and chronic thromboembolic pulmonary hypertension, respectively,^6,7^ causing poor life quality and requiring additional medical attention, including prolonged period of anticoagulation therapy.

**Editorial, see p 899**

**Meet the First Author, see p 894**

Mechanisms of thrombus development in veins remain incompletely understood. Risk factors for DVT include those that cannot be avoided or changed (such as old age or cancer) and various medical conditions, such as obesity, hypertension, and atrial fibrillation.^8,9^ About 60% of patients, who develop DVT and PE after hospitalization or surgery, develop the disease within 90 days,^8^ demonstrating connection between previous invasive therapy and DVT formation.

From the pathogenetic point of view, factors triggering thrombosis in veins differ from those in arteries. Arterial thrombosis usually starts with atherosclerotic plaque rupture with exposure of the subendothelial adhesive proteins, to which platelets bind forming a relatively small platelet-rich thrombus. In contrast, in veins, large thrombi enriched by red blood cells develop without signs of endothelial denudation.^10^ DVT begins in venous valvular sinus, in which blood flow can become stagnant resulting in prolonged time of blood exchange in valves, or stasis, an element of the Virchow triad.^11^ In venous valves, hypoxia and elevated hematocrit have been observed, which may increase local thombogenicity.^12^ The role of blood flow stagnancy increases in people in bed-ridden position caused by surgery or other situations accompanied by total or partial immobilization (limb paralysis, long-haul flights, etc).

We and others have demonstrated that an inflammatory component plays a role in triggering DVT.^13,14^ Local hypoxia may result in release of Weibel–Palade body (WPB) constituents, such as von Willebrand factor (vWF) and P-selectin, to the endothelial surface leading to recruitment of platelets and leukocytes.^15^ Platelets can contribute to thrombosis initiation likely by providing their procoagulant surface to clotting factors, whereas neutrophils support DVT propagation by producing neutrophil extracellular traps.^16,17^

Mast cells (MCs) are a part of the innate immune system. They originate from the hematopoietic progenitor in bone marrow and enter the circulation as early lineage progenitors.^18^ After egression to tissues and maturation, MCs express a specific set of antigens including FcεRI (a receptor for immunoglobulin E) and Kit (a receptor for stem cell factor). The role of MCs in allergic inflammation, a risk factor for DVT and PE,^19^ has been well documented.^20^ MC granules contain potent anticoagulants, such as heparin and tPA (tissue-type plasminogen activator); endothelial activators (histamine and tumor necrosis factor-α); and many enzymes (tryptases, chymases, and others).^21,22^ In tissues, MCs are located in the vicinity of blood vessels,^23^ and their presence at the site of human DVT has been reported.^24^

Herein, using murine models, we demonstrate that liberation of MC granule constituents is critical for DVT initiation. This is a novel mechanism regulating venous thrombosis, which could potentially be used to target DVT in humans.

## Methods

### Animals

MC-deficient Kit^W-v^ (Jackson Laboratory 000049, backcrossed to C57BL/6J for decades) mice^25^ and Kit^W-sh^ (Jackson Laboratory 012861, backcrossed to C57BL/6J for 11 generations) mice^26,27^ were purchased in the Jackson Laboratory, and colonies were maintained in animal facilities at Children’s Hospital Boston and the University of Birmingham under standard conditions. Wild-type littermates were used as a control in DVT and intravital microscopy experiments. C57BL/6 mice from Charles River were used in experiments with topical application of histamine and compound 48/80. All animal experiments were approved by either Institutional Animal Review Board at Children’s Hospital Boston or Animal Welfare Ethical Review Body and the UK Home Office (United Kingdom; Project Licenses 40/3745 and 70/8286).

### DVT Surgery

The stenosis of the inferior vena cava (IVC) was performed as described previously.^13^ In brief, mice were anesthetized with isoflurane–oxygen mixture, midabdominal incision was performed, and intestines were exteriorized and soaked in warm saline. All the IVC side branches were ligated. The IVC was gently isolated from aorta, a 7-0 polypropylene suture was placed over the IVC, and ligated over a spacer (30-gauge needle), and then, the spacer was removed. This method produces IVC lumen area reduction by ≈90% and is not accompanied by endothelial denudation. Finally, peritoneum was closed with silk suture and skin with either suture or staples. Thrombosis was evaluated at different time points ≤48 hours, and thrombi were excised for measurement. Assessment of thrombus weight and length by scale and ruler, respectively, has certain limitations; for example, precision of length measurement is ≈0.5 mm, whereas weight determination may vary ±1 to 1.5 mg. However, area of thrombi measured by ultrasonography and thrombus weight determined by scale demonstrate strong correlation (*r*^2^=0.96)^28^; and therefore, the manual method of thrombus size assessment used in this study can be considered valid.

### Culture of Murine Bone Marrow–Derived MCs

Bone marrow–derived MC culture was established as described elsewhere.^29^ In brief, bone marrow cells were flushed from adult wild-type (WT) mice femurs and tibia, centrifuged, and resuspended in RPMI (Roswell Park Memorial Institute) medium containing 10% fetal bovine serum, 100 U/mL penicillin, 0.1 mg/mL streptomycin, 25 mmol/L HEPES, 2 mmol/L l-glutamine, 1 mmol/L sodium pyruvate, 1 mmol/L nonessential amino acids, and 1 mmol/L MEM (minimal essential medium) amino acids (all from Sigma) in the presence of 5 ng/mL recombinant mouse interleukin-3 (Peprotech). A selection process for mature MCs was for a period of at least 4 weeks with continuous enrichment for nonadherent fraction of MC precursors. After this period, cells were phenotypically >90% mature as assessed by fluorescence-activated cell sorter staining with antibodies against mouse FcεR1-PE (eBioscience; clone MAR-1) and CD117(c-Kit)-APC (eBioscience; clone 2B8; Online Figure I). To induce MC degranulation, cells were sensitized overnight with 100 ng/mL mouse anti-DNP-IgE (Sigma), washed, and stimulated with 100 ng/mL DNP-HSA (dinitrophenyl-human serum albumin; Sigma) for 0.5 or 1 hour (fast release) or 6 hours (slow release). The efficiency of mediator release was monitored by β-hexaminidase activity in MC supernatants (releasates; Online Figure I).^30^

### MC Granule Depletion

Mice were pretreated IP every 12 hours for 4 days with the MC-degranulating compound 48/80 dissolved in 200 μL of sterile saline according to the following scheme: first day, 0.6 mg/kg; second day, 1.0 mg/kg; third day, 1.2 mg/kg; fourth day, 2.4 mg/kg. This treatment results in peritoneal MC granule content depletion by ≈84%.^31^ Twenty-four hours after last injection, mice were subjected to IVC stenosis for 48 hours.

### MC Membrane Stabilization In Vivo

MC membrane stabilizer sodium cromoglycate (cromolyn, 100 mg/kg body weight; Sigma) or 200 μL of sterile saline was injected IP into mice 24 hours and 30 minutes before surgery. Then, IVC stenosis was applied for 48 hours. Ketotifen (25 mg/kg body weight; Sigma) or 200 μL of sterile saline was injected IP 24 hours and 30 minutes before and 24 hours after IVC stenosis application for 48 hours. Sodium cromoglycate inhibits MC degranulation and has previously been shown to work in mice in the indicated or lower dose.^32,33^

### Murine Doppler Ultrasound

Doppler ultrasound was performed as described previously.^34,35^ Mice were anesthetized with isoflurane 2% plus oxygen, the region of interest was shaved, and covered with ultrasound gel. Images of the vena cava inferior and surrounding structures and vessels were obtained using the Visualsonics Vevo 2100 system with a cardiovascular scanhead.

### Determination of Plasma vWF Levels

The test was performed as described previously.^13^ The level of vWF in pooled plasma of 20 C57BL/6 mice served as a control.

## Results

### MC-Deficient Mice Are Protected Against DVT

**Figure 1.**
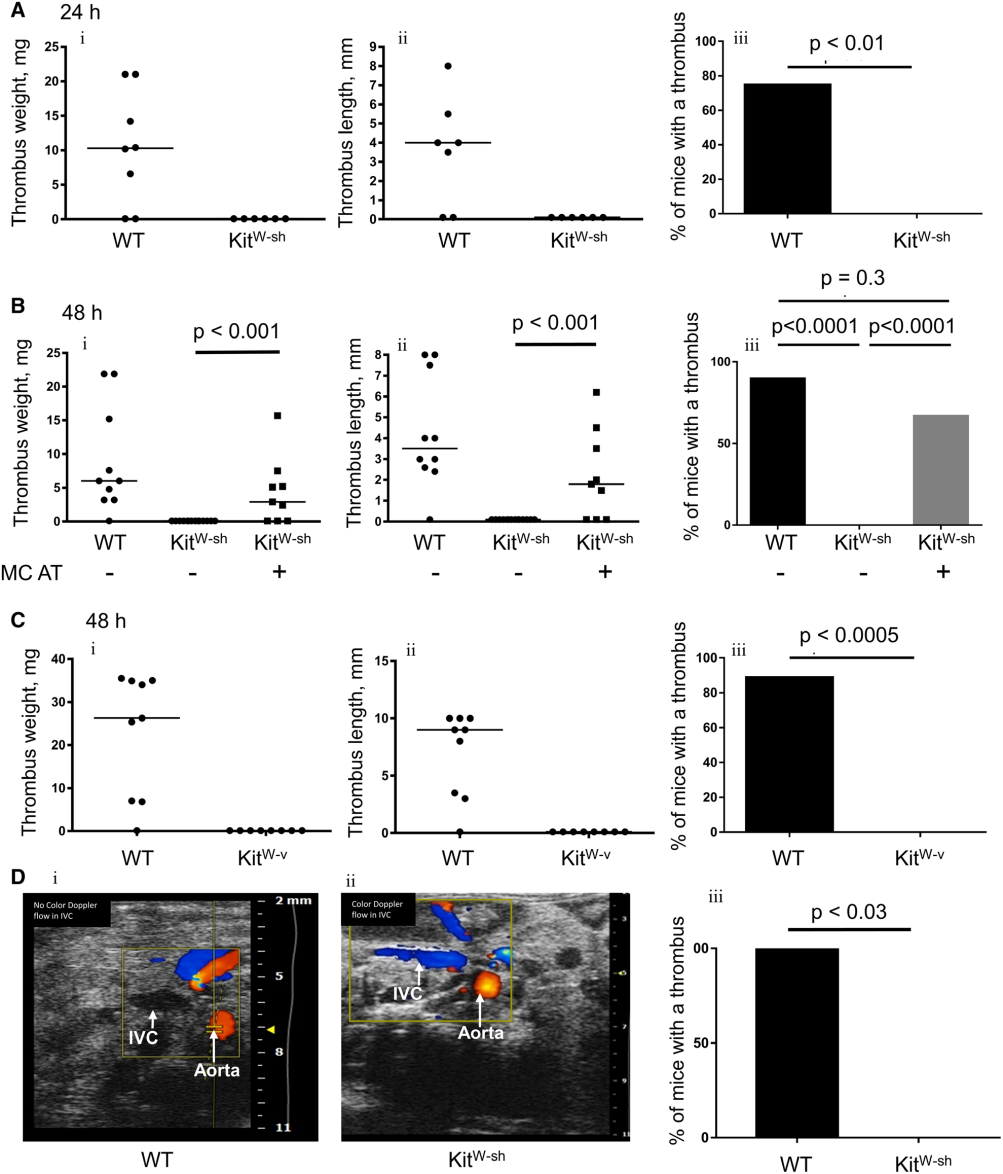
**Kit^W-sh^ and Kit^W-v^ mice are protected against deep vein thrombosis, and mast cell (MC) repopulation restores thrombosis.** Kit^W-sh^ mice and wild-type (WT) littermates were subjected to 24 h (**A**) or 48 h (**B** and **C**) stenosis of the inferior vena cava (IVC). A separate group of Kit^W-sh^ mice (n=9) had undergone adoptive transfer (AT) of 10×10^6^ in vitro differentiated MCs 3 mo before the experiment. **A**, WT, n=8; Kit^W-sh^, n=6. **B**, WT, n=10; Kit^W-sh^, n=14, Kit^W-sh^+AT, n=9. **C**, Kit^W-v^ mice (n=8) and WT littermates (n=9) underwent 48 h of the IVC stenosis. Presented are thrombus weight (i), thrombus length (ii), and thrombosis prevalence (iii). Note absence of thrombosis in both strains of MC-deficient mice and restoration of thrombosis after the adoptive transfer. **D**, WT (i) or a Kit^W-sh^ (ii) mice underwent IVC stenosis for 48 h, and then, thrombus formation was analyzed by Doppler ultrasound. Note lack of flow in the IVC of the WT mouse and presence of flow in the IVC of the MC-deficient mouse. (iii) Comparison of thrombosis prevalence between WT and MC-deficient mice analyzed by ultrasound.

To clarify whether MCs play a role in DVT, we subjected mice with W-sh Kit mutation, which lack MCs, to IVC stenosis. Both 24 and 48 hours after stenosis application, none of the mutant mice developed a thrombus compared with 75% and 90% thrombosis in WT controls, respectively (Figure 1A and 1B). To confirm the phenotype, we used another strain of Kit mutants, Kit^W-v^, with littermate controls. After 48-hour stenosis, 89% of control mice developed a thrombus, whereas no thrombosis was observed in Kit^W-v^ animals (Figure 1C). Further experiments were performed using Kit^W-sh^ mutants with littermate controls. To verify presence of thrombi in WT and absence in Kit^W-sh^ mice by an independent method, we used ultrasound Doppler (Figure 1D). Control WT mice with a thrombus did not have blood flow in the IVC and their IVC could not be compressed by applying pressure on the mouse abdomen with the probe. In contrast, Kit^W-sh^ mice had clear blood flow in the IVC, and the IVC was easily compressible suggesting absence of thrombi. The protective effect of MC deficiency was specific to DVT because no difference in thrombosis between WT and Kit^W-sh^ mice in ferric chloride–induced thrombosis was observed (Online Figure IIA). This finding indicates that the prothrombotic effect of MCs requires endothelial monolayer, which is denuded by ferric chloride.^36^ Tail bleeding time and coagulation tests (prothrombin time and activated partial thromboplastin time) in Kit^W-sh^ mice were unchanged compared with WT controls (Online Figure IIB through IID).

### MC Reconstitution Restores Thrombosis in MC-Deficient Mice

In addition to the absence of MCs, Kit mutation mediates other abnormalities, such as defective melanogenesis and impaired germ cell development. To rule out any other potential mechanisms, through which mutant Kit could affect thrombosis, we performed adoptive transfer of in vitro differentiated bone marrow MCs to Kit^W-sh^ mice, a procedure that restores MC pool in murine tissues.^37,38^ About 8 to 10 weeks after adoptive transfer, mice were subjected to IVC stenosis. As seen in Figure 1B, 67% of these animals produced a thrombus, which was significantly higher than MC-deficient mice without adoptive transfer and did not differ from thrombosis prevalence in WT controls. Thrombus weight and length in Kit^W-sh^ mice with and without adoptive transfer were also significantly different. Thus, it is the lack of MCs that protects against DVT.

### Granule-Containing MCs Are Present in the IVC Wall and Their Number Decreases With Thrombosis

**Figure 2.**
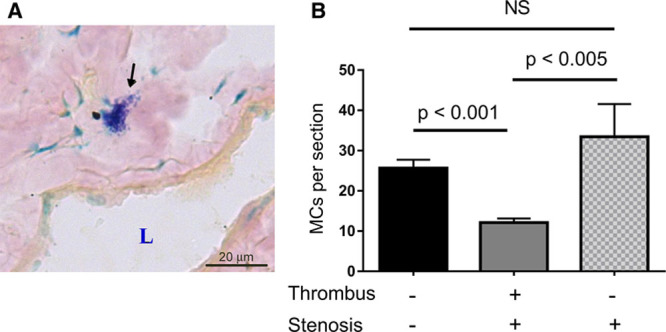
**Granule-containing mast cells (MCs) are present in the inferior vena cava (IVC) wall, and their number decreases with thrombosis.** **A**, IVCs from wild-type mice were frozen-sectioned and stained with Toluidine blue. MC is indicated by arrow. L, lumen. **B**, MCs were counted in transverse sections of intact IVCs (black bar, n=11), IVCs after 48 h stenosis with a thrombus (gray bar, n=11), and IVCs after 48 h stenosis that did not result in thrombus formation (patterned bar, n=7).

To test MC availability in the IVC, we performed staining of the vessel wall with Toluidine blue, a dye that specifically stains MC granules. MCs were found in the IVC wall in the vicinity to the abluminal side of the endothelium (Figure 2A). Forty-eight hours after stenosis application, the number of MCs in the IVC wall 2 to 3 mm below the ligation decreased in mice that developed thrombi compared with sham-operated animals (12.1±1.0 versus 25.6±2.1; *P*<0.0001; Figure 2B; Online Figure III). This result suggests that in the course of DVT, MCs either migrate out of the vessel wall or degranulate and thus lose Toluidine blue granule staining. The number of MCs in the IVCs of mice after 48-hour stenosis but without thrombi remained unchanged (33.4±8.1; *P*=0.27). This finding implies that degranulation of MCs in the IVC wall is associated with thrombosis.

### MC Granule Depletion Prevents DVT

**Figure 3.**
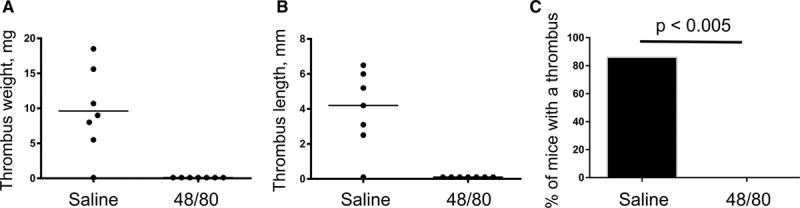
**Mast cell (MC) granule depletion prevents deep vein thrombosis.** MC granules were depleted by consecutive daily injections of the compound 48/80 as described in the Methods section. Control mice were administered sterile saline. Then, mice were subjected to 48 h inferior vena cava stenosis, and thrombi were excised and measured. n=7 in both groups. Presented are thrombus weight (**A**), thrombus length (**B**), and thrombosis prevalence (**C**).

We next assessed whether pharmacological ablation of MCs phenocopy the absence of DVT in Kit^W-sh^ mice. MC granules were depleted by consecutive daily injections of compound 48/80 as described in Methods section. Thrombi developed in 86% of control saline-treated mice, whereas none of the mice administered with compound 48/80 had a thrombus (Figure 3). This result implied that functional MCs are critical for venous thrombosis.

### Lack of MCs Is Accompanied by Suppressed Endothelial Activation

**Figure 4.**
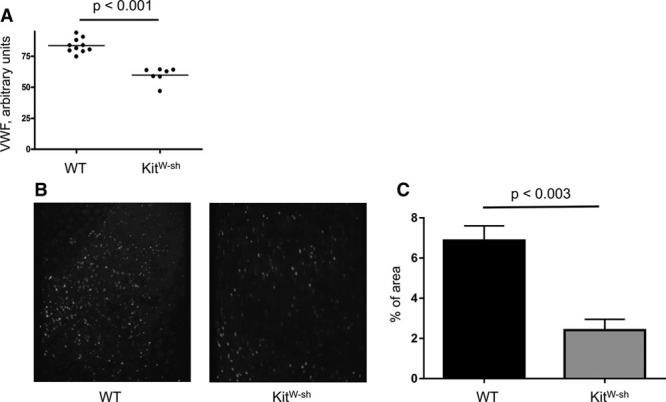
**Mast cells regulate plasma levels of von Willebrand Factor (vWF) and platelet recruitment to the stenosed inferior vena cava (IVC) wall.** **A**, Blood was drawn from the retro-orbital plexus of unchallenged wild-type (WT) and Kit^W-sh^ mice (n=7–10), stabilized with sodium citrate, and plasma levels of vWF were measured. **B**, Representative composite images of 10 consecutive frames of intravital microscopy showing fluorescent platelets adhered to the IVC wall 6 h after stenosis induction. Only mice without a thrombus at this time point were used. **C**, Percent of the area covered by adhered platelets in WT (black bar) and Kit^W-sh^ mice 6 h after stenosis application. n=4 for both groups.

We have previously reported the key role of endothelial activation and secretion of WPB constituents for DVT initiation in mice.^13^ Because mice lacking MCs are protected against DVT, we next tested whether MCs regulate activation of the endothelium. Unchallenged Kit^w-sh^ mice had lower plasma levels of vWF (Figure 4A), suggesting an association between presence or absence of MCs and circulating levels of vWF under quiescent conditions. Stenosis of the IVC promotes cell recruitment, which is a prerequisite to thrombus development.^13,17^ Therefore, we next tested whether this process is modified in the absence of MCs. Using intravital microscopy, we demonstrated that in WT mice 6 hours after stenosis application, multiple platelets stay adherent to the IVC wall (Figure 4B; Online Movie I). In contrast, in MC-null mice, only single platelets were adhered with majority of the cells moving with blood flow without detectable contact with the endothelium (Figure 4B; Online Movie II). The area covered by adhered platelets was 6.9±0.73% in WT mice versus 2.4±0.54% in Kit^W-sh^ animals (*P*<0.003; Figure 4C). This result suggests that MCs are implicated in endothelial activation preceding DVT. MC releasate was also able to stimulate expression of ICAM-1 (intercellular adhesion molecule-1) on HUVECs (human umbilical vein endothelial cells) in vitro (Online Figure IV), which is involved in leukocyte accumulation near the vessel wall. In addition, stenosis induced elevation in plasma levels of sP-selectin, a biomarker of venous thrombosis both in experimental animals and patients,^17,39–42^ in control but not in Kit^W-sh^ mice (Online Figure V). Thus, MC deficiency results in suppressed cell recruitment to the vascular wall at early stages of DVT, potentially because of impaired release of adhesive substances from the endothelium.

### Prevention of MC Degranulation Is Protective Against DVT

**Figure 5.**
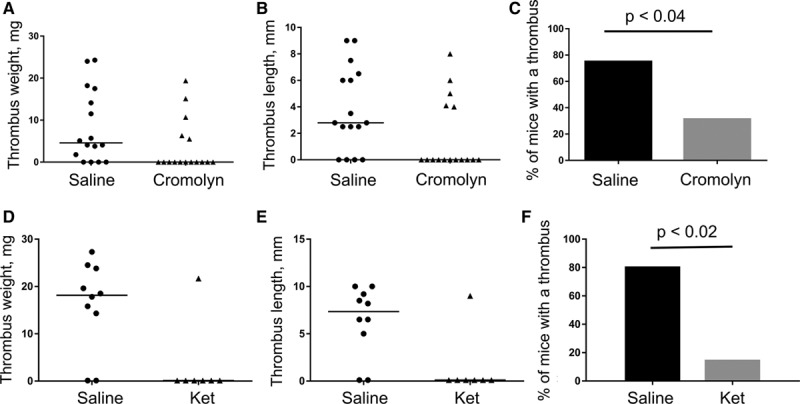
**Inhibition of mast cell degranulation by membrane stabilization protects against deep vein thrombosis (DVT).** **A**–**C**, Wild-type (WT) mice were administered sodium cromoglycate IP (cromolyn, 100 mg/kg body weight, n=17) or sterile saline (n=16) 24 h and 30 minutes before inferior vena cava stenosis induction. **D**–**F**, WT mice were injected with ketotifen IP (25 mg/kg body weight, n=7) or sterile saline (n=10) 24 h and 30 minutes before and 24 h after DVT surgery. Thrombus formation was checked 48 h later. Thrombus weight (**A** and **D**), thrombus length (**B** and **E**), and thrombosis prevalence (**C** and **F**) are shown.

MCs fulfill their biological functions largely through release of their granule components. Drugs stabilizing MC membrane preventing degranulation are on the market and used predominantly to treat allergic diseases. We next studied whether this pharmacological approach could be beneficial for DVT prophylaxis. Systemic administration of sodium cromoglycate (cromolyn) or ketotifen, MC membrane stabilizers, exerted an antithrombotic effect in the DVT model (Figure 5). Thrombosis prevalence fell from 71% in saline-treated controls to 31% in animals that received cromolyn (*P*<0.04) and from 80% to 14% in mice that were administered ketotifen (*P*<0.02). Thus, MC membrane stabilization can potentially be considered as an anti-DVT approach.

Topical application of compound 48/80 or histamine potentiates thrombosis in WT and induces DVT in Kit^W-sh^ mice.

**Figure 6.**
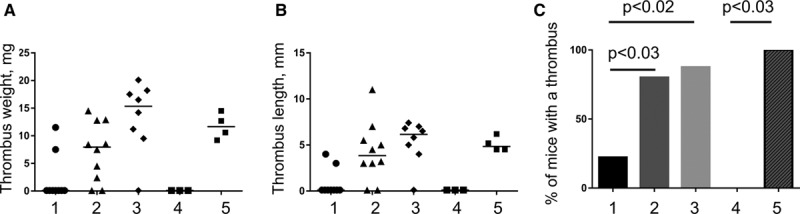
**Topical application of compound 48/80 or histamine potentiates thrombosis in wild type (WT) and histamine induces deep vein thrombosis in Kit^W-sh^ mice**. WT and Kit^W-sh^ mice underwent inferior vena cava (IVC) stenosis. During surgery, a strip of paper soaked in saline or solutions of either compound 48/80 (in WT mice only) or histamine (in both WT and Kit^W-sh^ mice) was applied to the IVC for 10 min immediately after stenosis induction. In 6 h, mice were euthanized, and thrombus formation tested. The groups are: 1, WT+saline; 2, WT+48/80; 3, WT+histamine; 4, Kit^W-sh^+saline; and 5, Kit^W-sh^+histamine. Presented are thrombus weight (**A**), thrombus length (**B**), and thrombosis prevalence (**C**).

MCs exacerbate DVT apparently by inducing endothelial activation and release of WPB. To further explore mechanisms, through which MCs exert their prothrombotic effect, we performed topical application of MC secretagogue compound 48/80 or histamine, a potent secretagogue of WPB uniquely synthetized and stored in MCs. Six hours of IVC stenosis resulted in thrombosis in 2 out of 8 (25%) WT control mice (Figure 6). In contrast, WT mice locally treated with compound 48/80 developed thrombus in 80% of cases (*P*<0.03). Histamine-treated WT mice developed a thrombus in 87.5% of cases (*P*<0.02 versus saline-treated animals). Interestingly, all Kit^W-sh^ animals, normally completely protected against DVT even at longer time points (48 hours), had thrombi after topical application of histamine, indicating that histamine can correct DVT defect in MC-deficient mice. Thus, it seems that local activation of MCs in WT mice promotes DVT, and this effect is likely mediated by histamine release.

We next performed administration of histamine H1 receptor (which is expressed on endothelial cells^43,44^) antagonists, pyrilamine maleate or cetirizine, to test whether inhibition of histamine effect can inhibit thrombosis. No protective effect of either of the substances on DVT was observed (Online Figure VI).

## Discussion

Current methods of DVT prophylaxis, targeting clotting factors, are inevitably accompanied by bleeding complications in a certain percent of patients. This is not surprising because components of blood coagulation system are involved in normal hemostasis, and downregulation of their function predictably affects physiological bleeding arrest. We report here (1) a novel role of MCs in DVT and (2) an attempt to prevent venous thrombosis using an alternative approach—inhibition of MCs, which are not directly implicated in hemostasis and therefore targeting them is unlikely to cause pathological bleeding.

Venous thrombus formation induced by flow distortion is based on endothelial activation, release of WPB components, and recruitment of innate immune cells and platelets, which precede thrombosis.^13,17^ Prevention of recruitment of immune cell, for example, in P-selectin–deficient mice, or platelets, results in strong protection against DVT.^13,17^ Thus, mechanisms of DVT in this model include not only blood coagulation but also elements of sterile inflammation. MCs contain both inhibitors of coagulation, such as heparin and tPA, which could be expected to inhibit thrombosis, and proinflammatory mediators, such as histamine and tumor necrosis factor-α, which could exert a prothrombotic effect given that DVT is an immunothrombosis and its mechanisms involve inflammatory components.^45^ This made it initially difficult to predict which of the pathways would turn out to be more important for DVT. We demonstrate that 2 independent strains of MC-deficient mice were completely protected against DVT in the IVC stenosis model. Lack of thrombosis in the absence of MCs suggests that MCs contain an entity, whose prothrombotic potential outweighs the antithrombotic power of one or several other MC components. One potential explanation of the relative inactivity of MC-derived anticoagulants is that MCs are located in the tissue surrounding the IVC, and their released granule constituents may reach abluminal part of endothelium but not the blood stream. In this scenario, proinflammatory mediators can activate endothelium, whereas heparin and tPA may not exert their anticoagulant effect. It should be noted that a small proportion of WT mice may not produce thrombi in this model. This suggests that the prothrombotic activity of MCs is not absolute and because of unclear reason might not suffice for thrombus formation in certain cases. Also, it should be noted that our results have been obtained on a mouse model, which has substantial differences from the human situation. In particular, murine IVC does not contain valves, whereas in humans, DVT develops inside venous valves. Also, humans have vertical spinal orientation, whereas mice have horizontal one, which means importance of muscle pump in venous return of blood to the heart in humans and unimportance of it in mice. There are difference also in various aspects of the immune system.^46^ For example, neutrophils constitute different percent of leukocytes in mice in humans,^46,47^ which might be relevant to venous thrombosis as neutrophils support DVT by expelling neutrophil extracellular traps.^16^ Thus, the validity of our results for humans remains to be verified in future translational studies in patients. The availability of MC membrane stabilizers on the market makes such studies possible in the not too distant future.

Mice used in this study lack MCs because of different mutations in c-Kit (CD117), a receptor to stem cell factor. In addition to absence of MCs, mutations in c-Kit may lead to other abnormalities. For example, homozygous Kit^W-v^ mice have macrocytic anemia and germ cell developmental defects, whereas Kit^W-sh^ mutation is accompanied by abnormal vascular permeability induced by bee venom phospholipase A2. Other still unknown MC-unrelated defects also cannot be excluded. To rule out alternative explanations of the phenotype in these mice, other than lack of MCs, we performed adoptive transfer of in vitro differentiated MCs into MC-deficient Kit^W-sh^ mice. This procedure has been shown to restore pool of MCs in different organs within 2 to 3 months.^37,38^ Adoptive transfer of MCs restored thrombosis in Kit^W-sh^ mice. Thus, the antithrombotic phenotype results primarily from absence of MCs and not from other defect caused by c-Kit mutation. Although engraftment of transferred MCs might differ from normal MC distribution and pattern of MC pool restoration in the IVC wall is unclear, such abnormal engraftment could be a potential explanation in case of lack of the phenotype recovery rather than in case of successful restoration of thrombosis by MC infusion.

Connective tissue MCs are usually located near blood vessels.^48^ The IVC turned out to be no exception as MCs were found in its wall in the vicinity to abluminal part of the endothelium. Induction of stenosis, which eventually resulted in thrombus formation, was associated with decreased numbers of granule-containing (toluidine blue-positive) MCs per section, whereas MC quantities remained unchanged after stenosis that because of unknown reasons did not lead to thrombosis. This result suggests a link between MC function and DVT development. It is unclear though whether MCs migrate out of the IVC wall or fully degranulate becoming invisible in Toluidine blue staining. Disappearance of MCs specifically in thrombosed IVCs implies that their released cargo triggers DVT.

In addition to genetic mutations, MCs in mice can be targeted pharmacologically. Chronic administration of MC secretagogue, compound 48/80, makes MCs temporarily functionally inactive because of exhaustion of intracellular pools of mediators.^31^ A similar effect can be achieved by MC membrane stabilizers that prevent the liberation of MC granule content.^32^ We tested both approaches, and they protected mice from DVT. In case of compound 48/80, the antithrombotic effect might also be attributed to downregulation of coagulation factor VII activation,^49^ and, therefore, in addition to its effect on MCs, the compound may suppress also the coagulation cascade. Thrombin generation is important for DVT in this model because low-molecular-weight heparin prevents thrombosis.^17^ Partial but significant protection by inhibiting MC degranulation is of particular importance because multiple drugs of this kind are already on the market and used for antiallergic purposes. Sodium cromoglycate used as a MC stabilizer does not have an antiplatelet effect, as shown by platelet function analyzer-100,^50^ and, therefore, platelet inhibition as a mechanism of DVT prevention can be ruled out.

Thus, MCs maintain vWF secretion in unchallenged mice (Figure 4A) and exacerbate DVT likely by stimulating activation of the endothelium and promoting WPB release. Failure to recruit platelets in the intravital microscopy in mice lacking MCs supports this statement. Platelet recruitment is a prerequisite for DVT and is dependent on vWF release from WPBs.^13,17^ The phenotype of MC-deficient animals is similar to vWF knockout mice: in both Kit^W-sh^ and vWF knockout mice, platelet accrual at the site of future thrombus is decreased. Besides platelets, leukocytes are also recruited to the IVC wall after stenosis and facilitate DVT by releasing neutrophil extracellular traps.^16^ Also, MC releasate was able to stimulate expression of ICAM-1, a major adhesion receptor for leukocytes, on endothelial cells in vitro. Consequently, MCs upregulate endothelial activation and liberation of vWF and P-selectin from WPBs and by this support DVT.

Interestingly, IVC stenosis is accompanied by elevated plasma sP-selectin levels, which can originate from both platelets and endothelium. Regardless of sP-selectin origin in this particular model, its plasma content is also regulated by MCs because in Kit^W-sh^ mice no sP-selectin upregulation was observed. Plasma sP-selectin is known to promote DVT in mice and is a DVT biomarker^39,42,51^ so that its increase in WT mice after IVC stenosis further suggests the similarity between the animal model and the human disease.

Topical application of MC secretagogue compound 48/80 stimulated thrombosis, suggesting that it is a MC granule constituent, likely a small molecule that affects endothelium or can permeate into blood, which promotes DVT in this model. Histamine is the likely candidate for stimulating WPB release. It is a potent WPB secretagogue,^52^ and MCs are a unique source of it. Local application of histamine to the IVC accelerated thrombosis in WT mice and induced DVT in MC-deficient mice. Thus, histamine is able to overcome the effect of MC deficiency, suggesting that it is the important constituent of MC granules, which may mediate MC prothrombotic effect. However, systemic administration of histamine H1 receptor inhibitors did not protect mice from DVT. This may be explained in several ways. First, histamine may not be a decisive WPB component, and the prothrombotic effect of MCs may require combined action of ≥2 additional components (eg, tumor necrosis factor-α). Second, histamine may act through >1 receptor. For example, H2 receptor operates in some vascular beds^53^ so inhibition of only 1 receptor might be insufficient. Third, histamine from MC granules reaches endothelial cells from their basal side, and, therefore, its effect may not be fully abolished by the receptor inhibitors, which operate at the luminal side. Given relatively long time of the experiment (48 hours), insufficient circulation time of the inhibitors also cannot be ruled out.

If MC absence or suppression protects against thrombosis, a question may arise as to why mastocytosis does not lead to thrombus formation. For example, no thrombi in the skin vessels of urticaria pigmentosa patients have been described. The explanation of this phenomenon may be that when the number of MCs increases, the ratio between anticoagulants (heparin, tPA) and proinflammatory mediators (histamine, tumor necrosis factor-α) released from MCs shifts toward the anticoagulants, rendering the resulting phenotype rather normal or bleeding than prothrombotic.

In conclusion, we have demonstrated that MCs are potent regulators of DVT in mice. The effect of MCs is likely to be at least partially implemented through release of histamine, although other MC granule constituents may contribute as well. Kit^W-sh^ mice have normal bleeding time, implying that temporary switching off MCs should not result in excessive bleeding. Thus, MCs may be considered a promising target for DVT prevention in humans.

## Acknowledgments

We thank Nashitha Kabir for technical assistance with ultrasound Doppler. We also thank Dr P. Harrison and Dr C. Gardiner for their help with murine coagulation tests.

## Sources of Funding

The work was supported by the British Heart Foundation (PG/13/60/30406) and the University of Birmingham to A. Brill and by the National Institutes of Health (5R01HL102101) to D.D. Wagner.

## Disclosures

None.

## Supplementary Material

## Novelty and Significance

### What Is Known?

- Development of deep vein thrombosis (DVT) includes mechanisms similar to sterile inflammation.
- Mast cells (MCs) reside in the vicinity to blood vessels.
- MCs contain granular mediators affecting both thrombosis and inflammation.
