# Supplementary material for: Mast Cells Granular Contents Are Crucial for Deep Vein Thrombosis in Mice
Source: Circ Res. 2017 Jul 24;121(8):941–50. doi: 10.1161/CIRCRESAHA.117.311185 (PMC5623089; doi:10.1161/CIRCRESAHA.117.311185)
Supplement: Supplementary file 1 [file res-121-941-s001.pdf]

SUPPLEMENTAL MATERIAL

Detailed Methods

Adoptive transfer of MCs into Kit^{W-sh} mice. Ten millions of *in vitro* differentiated MCs were injected into 3-4 weeks old Kit^{W-sh} mice through the tail vein. In 8 – 10 weeks, mice were subjected to 48 h IVC stenosis.

Toluidine blue staining and microscopy for mast cells granule content. Frozen sections were submerged in absolute ethanol, washed with running water and subjected to staining in Toluidine blue (TB) solution for 1 min. The staining solution was prepared freshly from 5 ml stock of TB (1% TB (Sigma) in 70% ethanol) combined with 45 ml fresh 1% NaCl solution in water (pH 2.0-2.5). After staining sections were washed with running water, submerged in 95% and 100% ethanol, soaked in a clearant and mounted with hydrophobic mount. Images were taken by Axio Scan.Z1 (Zeiss).

Local application of compound 48-80 or histamine. After stenosis application, a strip of Whatman paper soaked in solution of compound 48-80 (1 mg/ml), histamine (100 mg/ml; both from Sigma) or saline (control) was placed on the IVC for 10 min. Then peritoneum and skin were closed and mice were euthanized in 6 h to test the development of thrombi. Due to manual fashion of the paper strip application small variation in experimental conditions might take place from mouse to mouse.

Intravital microscopy of the stenosed IVC site and evaluation of the space covered with immobilized platelets. Mice were subjected to IVC stenosis for 6 h. Animals that had thrombi, as judged by observation through the IVC wall and inability to squeeze the IVC with forceps, were excluded from the experiment. Thereafter, mice were anesthetized with tribromethanol and syngeneic washed platelets labeled by calcein AM (from Sigma; 2.5% of the total amount of circulating platelets) were infused intravenously. Then the IVC was exposed and a coverslip was placed on its top. Fluorescent platelets were visualized 1-2 mm below (in caudal direction) the ligation site using 3i VIVO-SDC confocal system with Yokogawa CSU-10 and Photometrics Evolve EMCCD camera on an Olympus BX61WI upright microscope with air objective x10. Focus was adjusted to the upper focal plane. Flow was recorded for at least 1 min. Fiji/ImageJ33 software was used to evaluate areas covered by adhered platelets. Median-based intensity projection from 10 randomly chosen consecutive acquired images (corresponding to 1 s) was employed to generate a single projected image. After automatic set of threshold, total area of adhered platelets was measured by a Fiji incorporated function “Analyze particles”.

Treatment with histamine H1 receptor inhibitors. WT mice were administered pyrylamine maleate or cetirizine (both 30 mg/kg) 24 h and 30 min prior to IVC stenosis application, and 24 h thereafter. In 48 h after surgery, thrombosis was visually verified and thrombi were excised for measurement.

ICAM-1 expression on HUVEC cells. HUVEC cells were purchased from Gibco (Life Technologies Corporation, Carlsbad, CA) and grown according to the supplier instructions. For stimulation experiments, low passage HUVEC cells were plated in 24 wells until they reached confluency. Mast cells were primed overnight with anti-DMB IgE and stimulated with DMB for 6 h. The cleared MC releasate was applied on HUVEC cells for 20 h. After stimulation, cells were collected by Accutase treatment and stained with anti-human ICAM-1 (CD54, clone HA58, eBioscience). Flow cytometry was performed using Accuri C6 cytometer (BD Biosciences, USA).

Measurement of soluble P-selectin (sP-selectin) plasma levels. The content of sP-selectin was determined using ELISA kit (R&D systems) in accordance to manufacturer's instructions.

Coagulation tests. Prothrombin time (PT) and activated partial thromboplastin time (aPTT) were performed on Sysmex CS5100 coagulometer. Prothrombin time reagent Thromborel S and aPTT reagent Actin FS were from Siemens.

Statistics. Non-parametric results (e.g., length and weight of thrombi) were compared by the Mann-Whitney test. Parametric data (e.g., area covered by adhered platelets) were compared using two-tailed unpaired Student's t-test or (in case of multiple comparisons) one-way ANOVA. Thrombosis prevalence was compared by contingency table followed by the Fisher's exact square method. Statistics was calculated using GraphPad Prism 7 (GraphPad Software, Inc. La Jolla, CA). Difference between experimental groups was considered significant at $p<0.05$.

Supplemental Figures

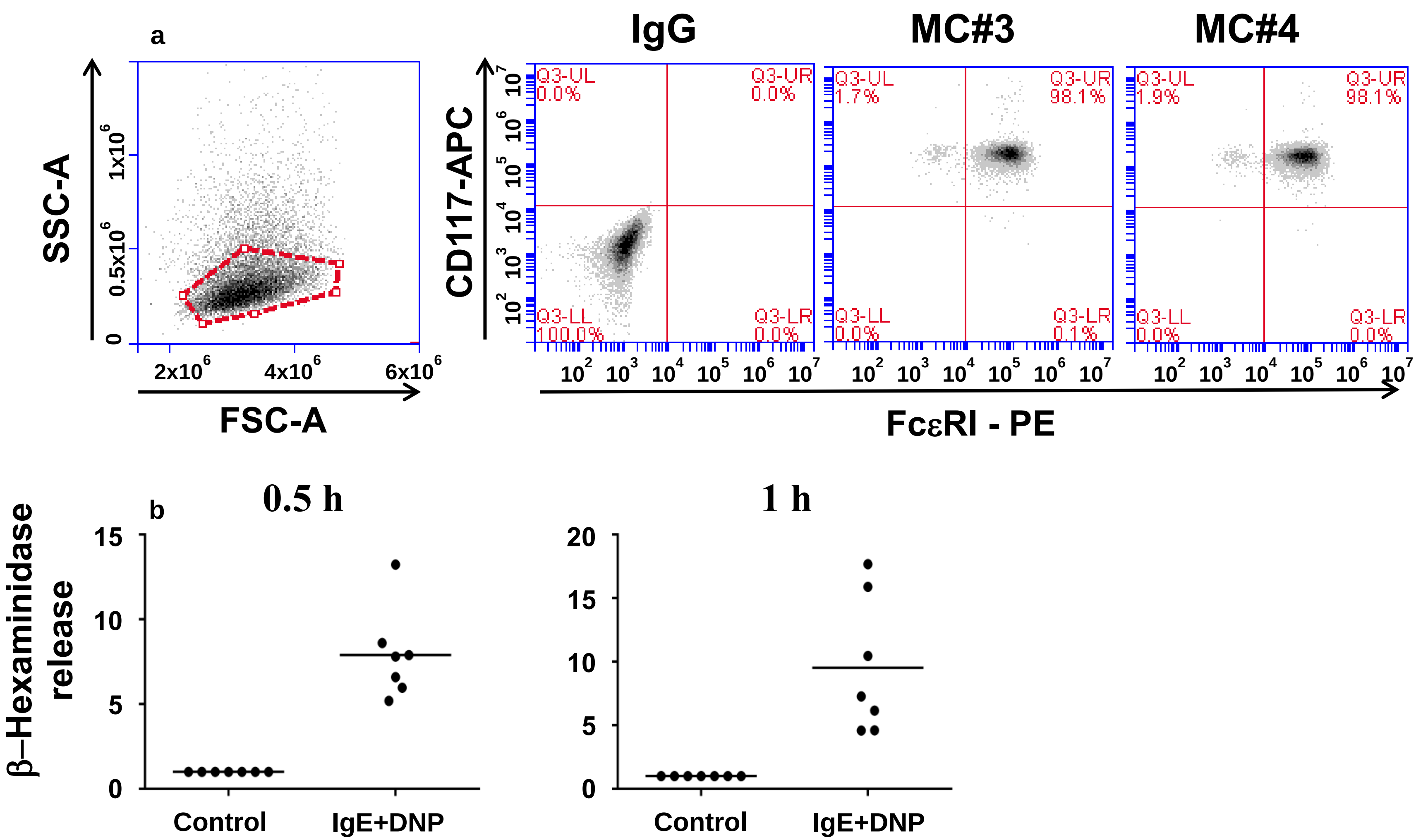

Online Figure I. Evaluation of purity and functionality of the in vitro differentiated MCs. (a) In vitro differentiated MCs were stained for CD117 and FcεRI; more than 90% of the cells expressed both receptors. Two MC batches used in the adoptive transfer experiments are presented (MC#3 and MC#4). (**b**) MC functionality was checked by their ability to secrete granular content upon stimulation. Each dot represents an individual cell line.

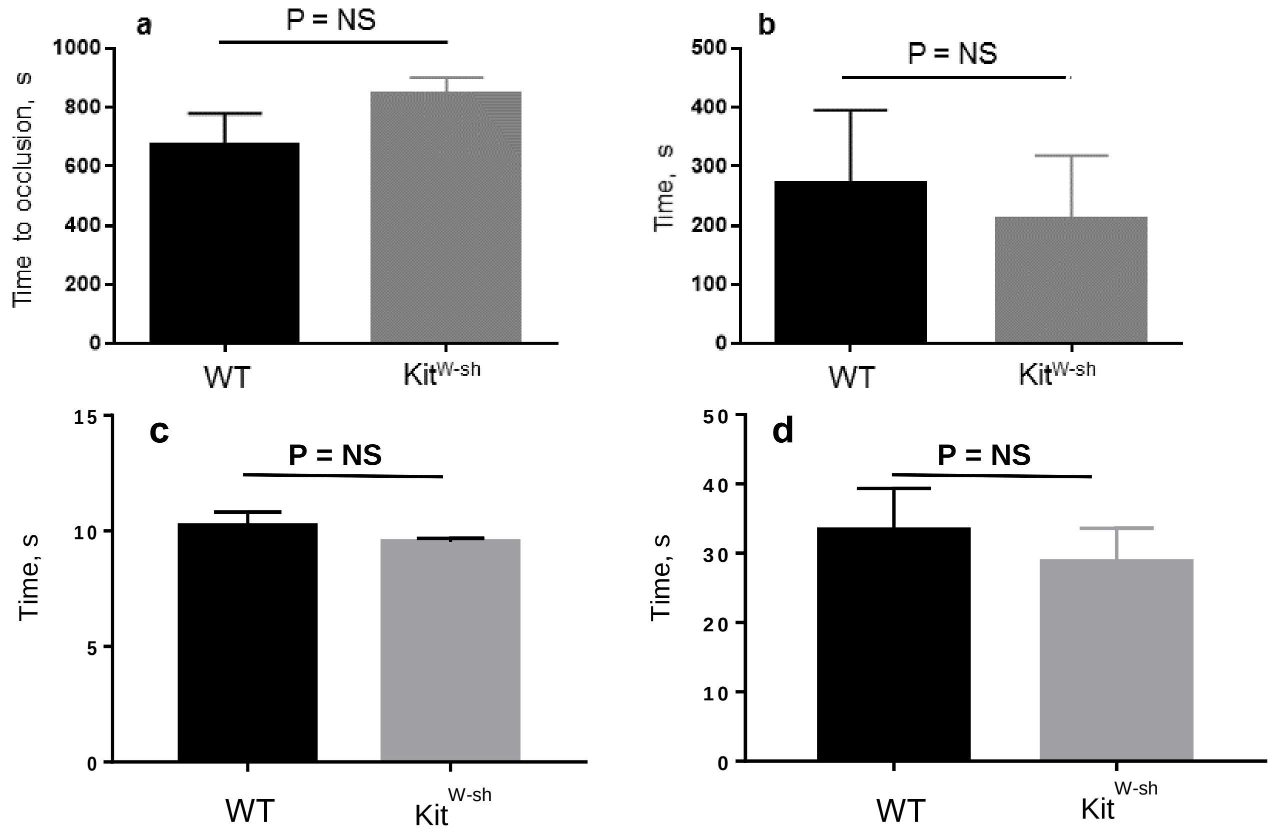

Online Figure II. No difference in normal hemostasis and arterial thrombosis between WT and Kit$^{W\text{-}sh}$ mice. (**a**) Fluorescent congenic platelets were infused into anesthetized WT or Kit$^{W\text{-}sh}$ mice (n=4 to 5 for each group), their mesentery was exposed and a strip of paper soaked in 10% ferric chloride was applied to the arteriole for 5 min. Time to full vessel occlusion was recorded (**b**) Tail tip (3 mm) was cut in anesthetized WT or Kit$^{W\text{-}sh}$ mice (n=6 to 8 for each group), placed into a test tube with warm saline and time to complete bleeding arrest was recorded. PT (**c**) and aPTT (**d**) tests were performed using a coagulometer (n = 4 to 5 mice per group).

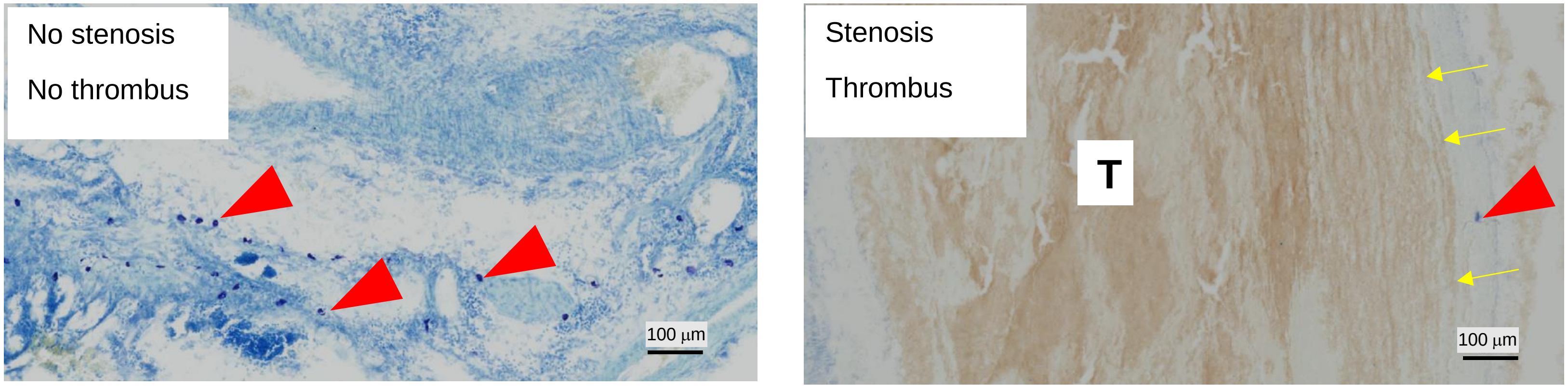

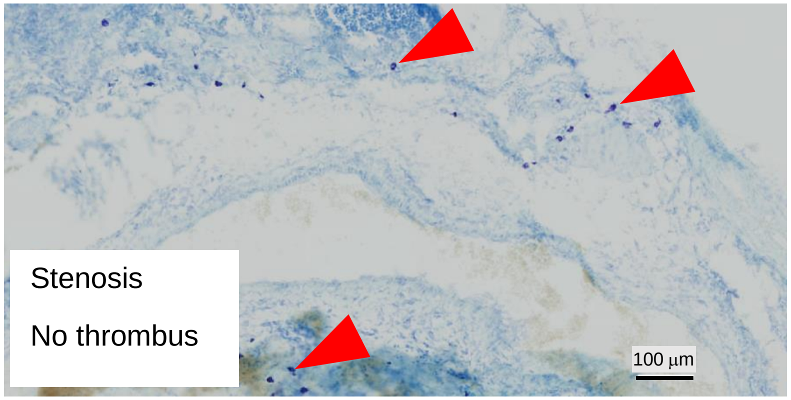

Online Figure III. Representative images of MCs in the vicinity of the IVC. IVC was excised from unchallenged mice (upper left) or mice after 48 h stenosis with (upper right) or without (upper right) a thrombus. Mast cells were stained with toluidine blue. Red arrow heads depict MCs, T designates thrombus. Yellow arrows show boarder between thrombus and the vessel wall.

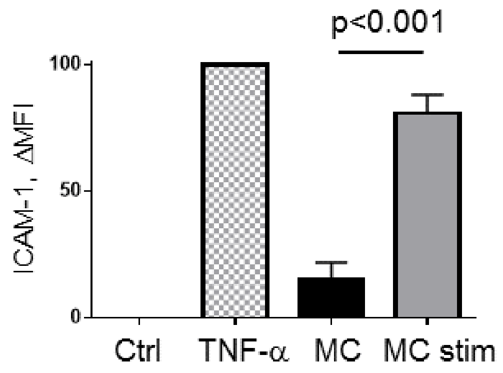

Online Figure IV. MC releasate induces ICAM-1 expression on HUVEC in vitro. In vitro differentiated MCs were stimulated with IgE followed by DNP as described in Methods and their releasate added to HUVEC for 20 h. Then, ICAM-1 expression was determined by FACS. ICAM-1 expression upregulation induced by 10 ng/ml TNF-α was taken as 100%.

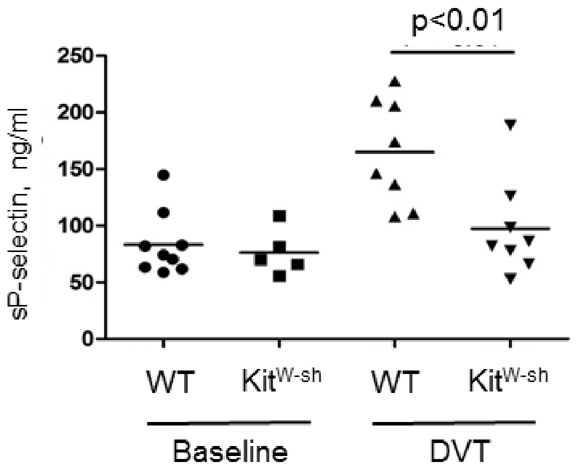

Online Figure V. DVT is accompanied by elevated plasma sP-selectin levels in WT but not MC-deficient mice. Blood was taken from retroorbital plexus of WT and Kit^{W-sh} mice (n=5 to 9) prior to surgery and 6 h after IVC stenosis application. Plasma levels of sP-selectin were determined using a commercial kit.

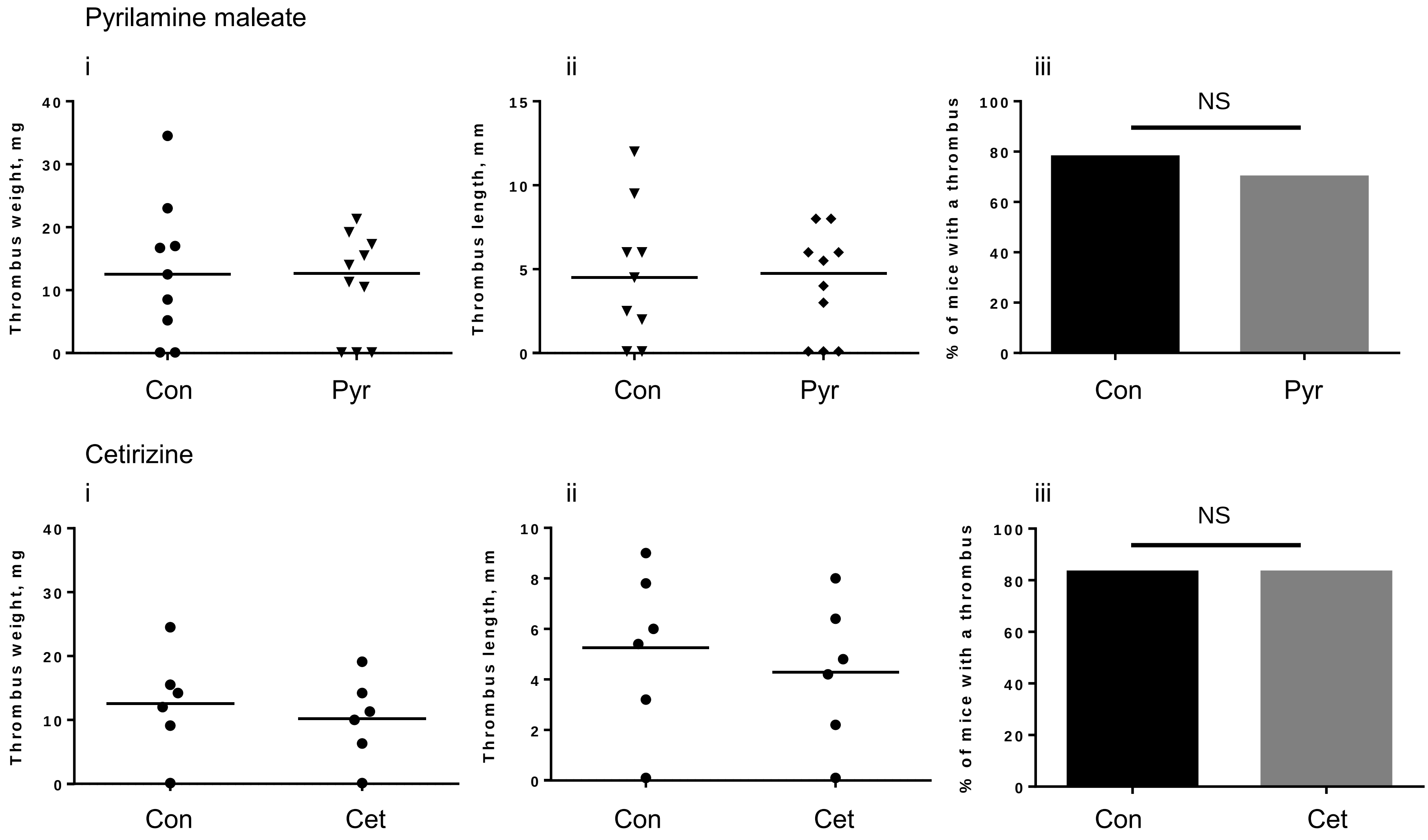

Online Figure VI. Systemic administration of histamine H1 antagonists does not protect against DVT. WT mice were injected pyrilamine maleate (upper row; con, n = 9, pyrilamine, n = 10), Cetirizine (lower row; n = 6 in both groups) or sterile saline as described in Methods, and then subjected to 48 h IVC stenosis. (i) Thrombus weight in mg, (ii) thrombus length in mm, (iii) thrombosis prevalence. Horizontal line in dot plots represents median. NS, non-significant.
